# Self-Care Practice and Associated Factors among Hypertensive Patients in Ethiopia: A Systematic Review and Meta-Analysis

**DOI:** 10.1155/2021/5582547

**Published:** 2021-04-09

**Authors:** Adam Wondmieneh, Getnet Gedefaw, Addisu Getie, Asmamaw Demis

**Affiliations:** ^1^Department of Nursing, College of Health Sciences, Woldia University, P.O. Box 400, Woldia, Ethiopia; ^2^Department of Midwifery, College of Health Sciences, Woldia University, P.O. Box 400, Woldia, Ethiopia

## Abstract

**Background:**

Hypertension is one of the leading causes of morbidity and mortality in developing countries including Ethiopia. Self-care practice has been provided as one of the most important preventive mechanisms of hypertension and is considered as a basic treatment for hypertension. There is no national-level study that assesses hypertensive self-care practice in Ethiopia. Therefore, this study aimed to assess the pooled level of hypertensive self-care practices and associated factors in Ethiopia.

**Methods:**

This study was carried out using published and unpublished articles accessed from databases: PubMed/MEDLINE, HENARI, Google Scholar, Web of Science, Scopus, African Journals, and university repositories. Data were extracted using a standard data extraction format. Data analysis was carried out using STATA version 11. Heterogeneity across the included studies was assessed using Cochrane's Q statistics and *I*^2^ test with its corresponding *p* values. Publication bias was determined using Egger's test and presented with a funnel plot. The pooled level of hypertensive self-care practice was estimated using a random-effects meta-analysis model.

**Results:**

This systematic review included 17 cross-sectional studies with 5,248 study participants. The overall pooled level of self-care practice among hypertensive patients in Ethiopia was 41.55% (95% CI 33.06, 50.05). Participant formal education (AOR = 2.82; 95% CI 2.18, 3.64) and good knowledge of hypertension (AOR = 4.04; 95% CI 2.19, 7.44) were significantly associated with self-care practice among hypertensive patients in Ethiopia.

**Conclusion:**

In this study, more than half of hypertensive patients had poor hypertensive self-care practice in Ethiopia. Participant's formal education and good knowledge of hypertension were significantly associated with self-care practice among people living with hypertension in Ethiopia. Therefore, based on the evidence of this study, we recommended that programmers and policymakers should enhance the awareness of hypertensive patients on self-care practice domains and strengthen local programs working on noncommunicable diseases.

## 1. Background

Hypertension is one of the leading causes of morbidity and mortality worldwide. In 2010, a total of 1.39 billion adult population worldwide had hypertension and 1.04 billion hypertensive people were from middle- and low-income countries [1]. The prevalence of hypertension among the adult African population aged 25 and above is the highest among other regions of the world (46%) [2]. In Ethiopia, the prevalence of hypertension was 19.6% [3]. In middle- and low-income countries including Ethiopia, the prevalence of hypertension significantly rises due to unhealthy dietary practices, the rapid expansion of urbanization, and the sedentary lifestyle of people [4–6]. People who are living in middle- and low-income countries are highly suffering from hypertension because of inadequate access to health services; low socioeconomic status of people; and low level of awareness, diagnosis, and treatment of hypertension [2, 7].

A systematic review and meta-analysis conducted in Ethiopia showed that 48% of hypertensive patients do not have their blood pressure controlled below the level of 140/90 mmHg [8], which indicates that hypertension is poorly managed in Ethiopia. Uncontrolled hypertension is associated with an increased risk of cardiovascular diseases, renal diseases, eye disease, and cerebrovascular complications [9, 10]. Globally, hypertension accounts for 9.4 million deaths each year. Moreover, hypertension is responsible for 45% of deaths from total ischemic heart disease mortality and 51% of deaths from total cerebrovascular disease mortality [2].

Several modifiable factors are associated with a high prevalence of hypertension. These factors are excessive alcohol consumption, smoking a cigarette, overweight and obesity, inadequate access to health services, consumption of high sodium diets, and nonadherence to antihypertensive medications [2, 11]. Evidence showed that excessive alcohol consumption was considerably associated with elevation of blood pressure [12, 13]. Therefore, reducing alcohol consumption favors blood pressure reduction [14]. Tobacco use increases the risk of cardiovascular complications among patients with hypertension. Additionally, hypertensive patients who smoke cigarettes are at high risk of developing a severe form of hypertension including malignant hypertension [15]. Globally, cigarette smoking contributes to six million annual deaths [11]. Therefore, cessation of smoking plays a significant role to prevent the complications of hypertension.

Performing regular physical activity for 30 minutes on 5–7 days per week may be helpful for the prevention as well as the treatment of hypertension [16]. Evidence suggested that a reduction of salt intake causes a significant reduction in blood pressure [17]. In overweight and obese hypertensive patients, weight loss is associated with the reduction of blood pressure [18]. Adherence to antihypertensive medications is crucial to reduce the life-threatening complications of hypertension. However, only 54.8% of hypertensive patients adhere to antihypertensive medications [19]. Nonadherence to antihypertensive medications was significantly associated with an increased risk of stroke and cardiovascular diseases [20, 21].

Self-care practice has been provided as one of the most important preventive mechanisms of hypertension and is considered as a basic treatment for hypertension [16]. Self-care practices are also recommended as adjuvant therapy for those hypertensive patients who take antihypertensive medications to enhance the antihypertensive effect [22, 23]. Hypertensive self-care practices mainly focused on weight reduction, cessation of smoking, moderation of alcohol consumption, performing regular exercise, salt reduction, and adherence to antihypertensive medications [16, 24]. The life-threatening complications of hypertension can be reduced by improving the self-care behaviors of hypertensive patients [16]. Identification of risk factors that influence self-care practice among hypertensive patients is essential to decrease the morbidity and mortality of hypertension. Hypertensive self-care practices can be affected by several factors such as gender, age, body mass index (BMI), educational status, monthly income, knowledge on hypertension, and presence of comorbidity [25, 26].

In Ethiopia, several single studies reported the level of hypertensive self-care practice in different regions, which ranges from 20.3% [27] to 68.92% [28]. However, there is no national-level study that estimates the overall level of self-care practice among hypertensive patients. Estimating the overall hypertensive self-care practice is very crucial for policymakers and planners to evaluate the implementation of hypertension treatment and prevention programs and to update the current self-care practice. Therefore, this study aimed to estimate the pooled level of self-care practice and associated factors among hypertensive patients in Ethiopia.

## 2. Materials and Methods

### 2.1. Databases and Search Strategy

Studies reporting the level of self-care practice and/or a variety of factors associated with hypertension self-care practice in Ethiopia were systematically searched from electronic databases: PubMed/MEDLINE, HENARI, Google Scholar, Web of Science, Scopus, African Journals, and university repositories. Additionally, the reference list of established articles was searched to identify additional literature. Studies reporting the level of self-care practice and/or recommended lifestyle among hypertensive patients in Ethiopia were included in the final meta-analysis. Searching was conducted using the following keywords: prevalence, adherence, hypertensive self-care, salt reduction, physical activity, cigarette smoking, alcohol intake, weight reduction, and medication adherence. The keywords were combined using “AND” and “OR” Boolean operators. The searching date was from November 01, 2020, to December 20, 2020, and articles accessed until December 25, 2020, were included in this systematic review and meta-analysis. The preferred reporting item for the systematic review and meta-analysis (PRISMA) guideline was used to report this systematic review and meta-analysis [29].

### 2.2. Eligibility Criteria

#### 2.2.1. Inclusion Criteria

Published and unpublished studies conducted only in Ethiopia and satisfying the following inclusion criteria were included in this systematic review and meta-analysis.

  Study design: observational studies (cross-sectional, cohort, and case-control)
  Language: only studies reported in the English language
  Study participants: studies were conducted among all hypertensive patients
  Publication year: articles published before December 25, 2020, were included in this study

#### 2.2.2. Exclusion Criteria

Articles that were inaccessible full text after two contacts with the primary author by e-mail were excluded. Additionally, interventional studies, systematic reviews, case reports, narrative reviews, and policy statements were excluded from this study.

#### 2.2.3. Outcome Measurement

In this study, hypertensive self-care practice were measured using six domains: regular physical activity, salt reduction, cessation of smoking, moderation of alcohol consumption, and weight reduction [16].

  Regular physical activity: participants performing regular physical activity ≥30 minutes per day, at least 4 days per week
  Salt reduction: reduce salt when preparing foods and avoid or limit the consumption of salty foods
  Smoking cessation: hypertensive patients who did not smoke any pack of cigarettes
  Moderation of alcohol consumption: daily alcohol consumption does not exceed 2 standard drinks per day for men and 1 standard drink per day for women (1 standard drink = 10 gram alcohol)
  Weight management: manage weight through dietary practice as well as performing exercise to lose weight
  Medication adherence: hypertensive patients took medication at the recommended dosage and time

### 2.3. Quality Assessment

In this study, the quality of the included studies was assessed using the Newcastle-Ottawa Quality Assessment Scale for the cross-sectional study [30]. The scale has three components. The first component mainly assessed the methodological quality of each study and graded from five points. The second component graded from two points and mainly focused on the comparability of studies. The third component of the tool focused on the outcomes and statistical analysis of each article and graded from three points. Two investigators (AW and GG) assessed the quality of each study and studies with a scale of six and above six out of 10 scales were determined as high quality.

### 2.4. Data Extraction

The data were extracted by three independent investigators (AW, AD, and GG) using a Microsoft Excel spreadsheet. The disagreement between the investigators was resolved by the fourth author (AG) through discussion. The data extraction format included information on author name, year of publication, the region of study, study design, sample size, response rate, self-care practice, mean age, sex, residence, predictors, and each domain of hypertensive self-care practice.

### 2.5. Data Processing and Analysis

The pertinent data were extracted and further cleaned using a standard data extraction format, prepared in Microsoft Excel sheet, and data were exported into STATA version 11.0 (Stata Corporation, College Station, Texas) for further analysis. The data were analyzed using Der Simonian and Laird's random effect meta-analysis model at 95% CI. Heterogeneity between the included studies was assessed using Cochrane Q statistics and *I*^2^ percentage with its corresponding *p*-values [31, 32]. In this study, the statistical value showed that there was high heterogeneity detected across the included studies (*I*^2^ = 97.4%, *p* ≤ 0.001). Therefore, a random-effects meta-analysis model was used to estimate the pooled level of hypertensive self-care practice in Ethiopia. Subgroup analysis was computed based on regions of the study, sample size, and year of publication to assess the possible source of heterogeneity detected in the pooled level of hypertensive self-care practice. Finally, the presence of publication bias was assessed using Egger's test [33] and presented graphically with funnel plots. A statistical test with a *p*-value <0.05 was considered statistically significant.

## 3. Results

### 3.1. Selection and Identification of Studies

**Figure 1:**
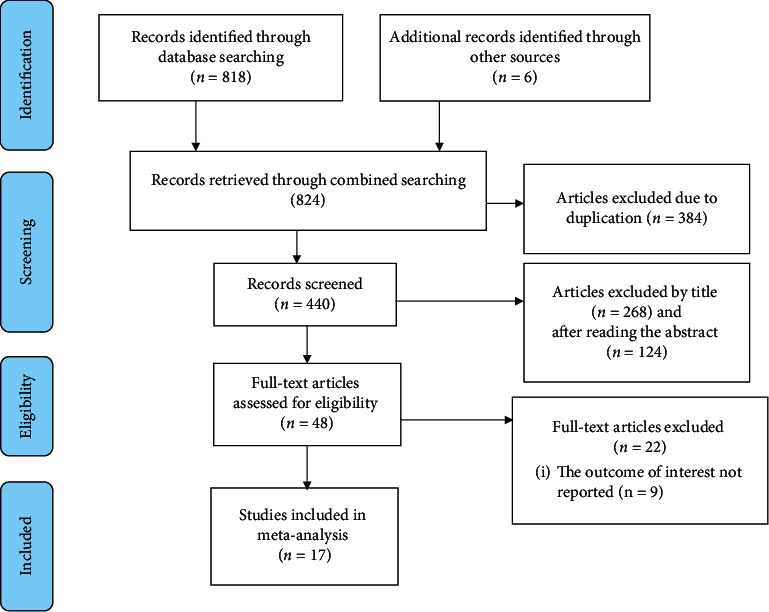
Flow chart of study selection for systematic review and meta-analysis to estimate the level of self-care practice among hypertensive patients in Ethiopia.

We collected 824 articles from the different databases of PubMed/MEDLINE, HENARI, Google Scholar, Web of Science, Scopus, African Journals, and university repositories and reference lists of established articles. From these studies, 384 duplicated records were removed. Among the remaining 440 studies, 392 irrelevant studies were excluded after assessing and screening titles and abstracts. Then, 48 full-text articles were assessed for eligibility and 31 articles were further excluded for being not in line with the inclusion criteria of our study. Finally, 17 studies fulfilled the inclusion criteria and were included in this systematic review and meta-analysis (Figure 1).

### 3.2. Characteristics of Included Studies

In this systematic review and meta-analysis, we included 17 cross-sectional studies with 5,248 study participants. All studies were published between 2016 and 2020 in peer-reviewed journals. The minimum and maximum sample size of the included studies were 130 [34] and 422 [35], respectively. The response rate was 98.41%. Half of the participants were male (50.4%) and the mean age of the participants was 54.55 years old. The majority of the participants are living in urban areas, 69.5%. The included studies were reported from five regions and one city administration of the country. Four studies were reported from the Oromia regional state [28, 34, 36, 37]. Three studies were from Amhara regional state [38–40], three studies from Harari regional state [35, 41, 42], three studies from Addis Ababa city administration [43–45], two studies from Tigray regional state [27, 46], and two studies from South Nations Nationalities and Peoples Regional State (SNNPR) [47, 48]. Four regions and one city administration, namely, the Afar region, Benishangul Gumuz region, Somali region, Gambella region, and Dire Dawa city administration, were not included in this study due to a lack of studies (Table 1).

### 3.3. Self-Care Practice among Hypertensive Patients in Ethiopia

**Figure 2:**
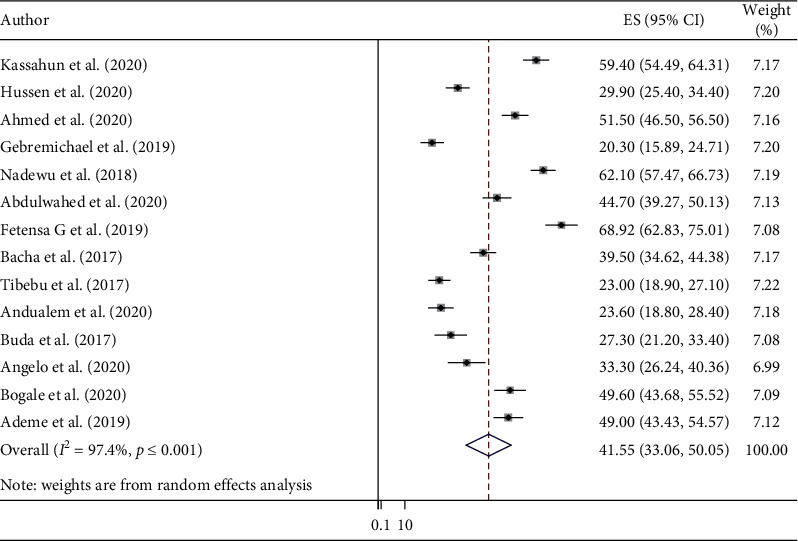
Forest plot of included studies that assess the level of self-care practice among hypertensive patients in Ethiopia.

From the total 17 included studies, 14 studies reported the level of hypertensive self-care practice in Ethiopia. The results of this meta-analysis showed that the overall estimated pooled level of self-care practice among hypertensive patients in Ethiopia was 41.55% (95% CI 33.06, 50.05) (Figure 2). The highest level of hypertensive self-care practice was reported from a study conducted in Nekemte (68.92%), Oromia region, 2019 [28]. In contrast, the lowest level of hypertensive self-care practice was reported from a study done in Ayder Comprehensive Specialized Hospital (20.3%), Tigray regional state, 2019 [27].

### 3.4. Subgroup Analysis

We conducted a subgroup analysis based on regions of the country where studies were conducted to investigate the possible source of heterogeneity. The results of subgroup analysis showed that the highest level of self-care practice among hypertensive patients was observed in studies conducted in Oromia regional state, 56.77 (95% CI 33.03, 80.58) (Table 2).

### 3.5. Adherence to Recommended Lifestyle Modifications among Hypertensive Patients in Ethiopia

In the current meta-analysis, we determined the level of hypertensive self-care practice using six lifestyle recommendations: physical activity, salt reduction, cessation of smoking, moderation of alcohol intake, weight reduction, and medication adherence. Accordingly, thirteen studies included information on the physical activity of hypertensive patients. In this meta-analysis, the pooled level of performing regular physical activity was 46.68 (35.03, 58.68). Similarly, thirteen studies reported cessation of cigarette smoking. The pooled level of cessation of smoking among hypertensive patients was 88.09 (83.99, 92.19) (Table 3).

### 3.6. Heterogeneity and Publication Bias

**Figure 3:**
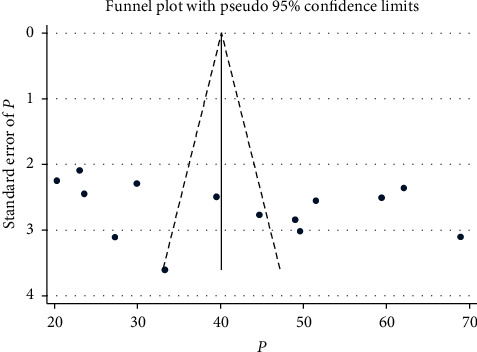
Funnel plot to assess the level of self-care practice among hypertensive patients in Ethiopia.

In this study, there was high heterogeneity across the included studies according to the Cochrane Q test and *I*^2^ test statistics (*I*^2^ = 97.4%, *p* ≤ 0.001) [49]. Therefore, a random-effects meta-analysis model was conducted to estimate the pooled level of self-care practice among hypertensive patients. The presence of publication bias was assessed using Egger's regression test and Begg's rank correlation test. The result of Egger's weight regression (*p* = 0.216) and Begg's rank correlation test (*p* = 0.189) suggested that there was no publication bias. The funnel plot of hypertension self-care practice in Ethiopia was symmetrical, suggesting no publication bias (Figure 3).

### 3.7. Factors Associated with Hypertension Self-Care Practice in Ethiopia

**Figure 4:**
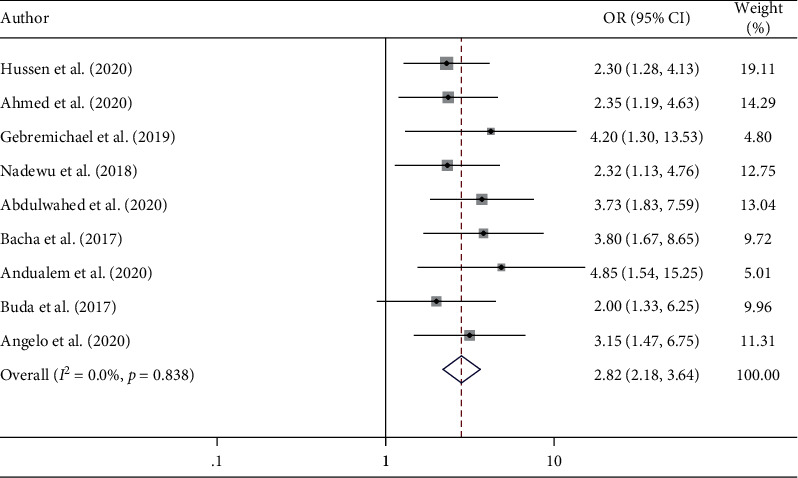
The pooled odds ratio of the association between educational status and self-care practice among hypertensive patients in Ethiopia.

**Figure 5:**
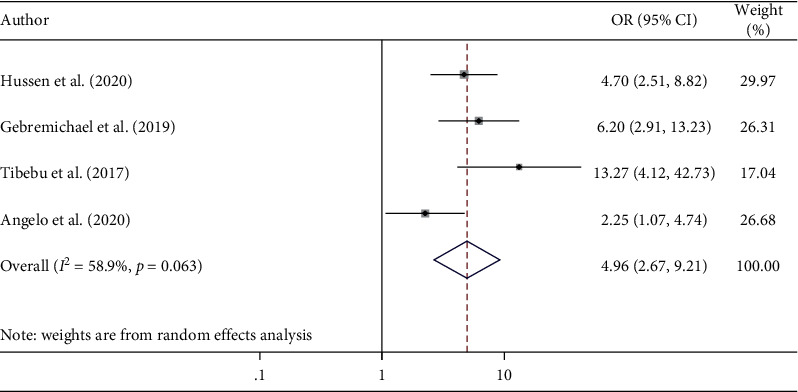
The pooled odds ratio of the association between hypertensive patient's knowledge and self-care practice among hypertensive patients in Ethiopia.

A total of nine cross-sectional studies have been included to determine factors significantly associated with hypertensive self-care practice in Ethiopia. The current study identified two predictors, namely, knowledge about hypertension and the educational status of hypertensive patients. The analysis of nine cross-sectional studies [27, 35, 37, 39, 41, 43, 44, 47, 48] showed that the educational status of the hypertensive patients was significantly associated with hypertensive self-care practice. Hypertensive patients who attended formal education were approximately 3 times more likely to adhere to hypertensive self-care practice than those who did not attend formal education (AOR = 2.82; 95% CI 2.18, 3.64) (Figure 4). The association between hypertensive patient's good knowledge and hypertensive self-care practice was assessed by four studies [27, 41, 45, 48]. The results revealed that hypertensive patients who had good knowledge of hypertension were 4 times more likely to practice self-care than those patients who had poor knowledge of hypertension (AOR = 4.04; 95% CI 2.19, 7.44) (Figure 5).

## 4. Discussion

Hypertensive self-care practice is very important for the prevention and treatment of hypertension [16]. As far as the investigator's knowledge, this study is the first of its kind to estimate the pooled level of hypertensive self-care practice in Ethiopia. Estimating the overall level of self-care practice among hypertensive patients is very crucial for hypertension treatment and prevention of complications related to hypertension. Adherence to the recommended hypertensive self-care practice requires continuous motivation and education from different stakeholders [14].

The findings of this study showed that more than half of hypertensive patients had poor hypertensive self-care practice in Ethiopia 41.55% (95% CI 33.06, 50.05). The current finding is in line with a study done in West Bagel, India (37.1%). However, this finding was lower than studies conducted in Ghana (72%) [50], Tertiary Level Hospital of Kathmandu, Nepal (57.1%) [51], and South India (60.6%) [52], and much higher than a study done in Nigeria (16.4%) [53]. The possible explanation for this variation might be due to a difference in socioeconomic status, cultural practices, educational status, access to health care facilities, and lifestyle of the study participants. Additionally, there was methodological variation and differences in the components of the hypertension self-care practice assessment tool.

In the current meta-analysis, more than half of the study participants have not performed regular physical activity 46.68 (95% CI: 35.03, 58.68). This finding is supported by a study done in Nepal (44.8%) [51] and America (52.2%) [54]. However, this finding is lower than a study done in Saudi Arabia (20.1%) [25]. This variation might be due to a lack of organized set up around the living areas for exercise and there are cultural differences. Aerobic exercise is an effective treatment for lowering blood pressure in hypertensive patients [55]. Therefore, performing a regular aerobic exercise for 30 minutes on 5–7 days per week may be important for both the prevention and treatment of hypertension [16].

In this meta-analysis, more than half of the hypertensive patients practiced the recommended salt reduction of 55.03% (95% CI: 45.58, 65.03). Evidence from the Cochrane library showed that dietary sodium intake reduction from 11.59 grams per day to 3.89 grams per day reduces blood pressure by 3% in hypertensive patients [56]. Reduction of the daily intake of salt by 5 grams decreases the risk of cardiovascular and stroke mortality by 17% and 23%, respectively [57]. The American College of Cardiology 2017 Guideline for the treatment and prevention of hypertension recommended that the daily intake of salt should not exceed 1.5 grams [58]. Therefore, the reduction of salt during food preparation and avoidance or limitation of consumption of salty foods may be beneficial for both in the reduction of mortality and morbidity in hypertensive patients.

In the current study, 47.9% (95% CI, 34.83 60.97) of hypertensive patients have practiced the recommended weight reduction. A similar finding was observed in a study conducted in Nepal (48.6%) [51]. A study conducted among African American hypertensive patients showed that only 30.1% of participants practiced weight reduction [54]. The current finding is much higher than a study conducted in India (11.4%) [59]. This variation could be explained by cultural differences. Additionally, there is methodological variation, in which this study analyzed the level of hypertensive self-care practices with a large sample size (5,248), while the above-mentioned studies were conducted at a specific place with a small sample size.

The results of this study showed that the majority of the study participants 88.09% (95% CI: 83.99, 92.19) were not smoking a cigarette. Similar findings were reported from a study done in Israel (87%) [60] and Nepal (84.9% [51]. The results of this meta-analysis showed that 11.91% of respondents still smoke a cigarette. A 15-year population-based cohort study conducted in Indonesia showed that cigarette smoking was significantly correlated with high blood pressure [61]. Therefore, cessation of smoking is crucial for the reduction of cardiovascular and cerebrovascular complications of hypertension [15].

In this meta-analysis, 67.26% (95% CI: 52.26, 82.26) of hypertensive patients were adherent to moderate alcohol intake. The result of this study is supported by a study conducted in the USA among African American study participants (65%) [54]. A cross-sectional study done in Nepal showed that 85.8% of study participants avoid alcohol consumption [51]. The American College of Cardiology recommended that daily alcohol intake should not exceed 2 drinks per day for men and one drink per day for women [58]. Reducing alcohol consumption lowers the blood pressure in people who drink more than two drinks per day [62]. Therefore, reducing alcohol intake is important to decrease the burden of hypertension.

In the current study, more than half of hypertensive patients 58.55% (95% CI: 50.26, 66.84) were adherent to antihypertensive medications. This finding is in line with a study conducted in India (54%) [59] and in America, 2011 (58.6%) [54]. However, this finding is lower than a study reported from America in 2015 (69%) [63] and Korea (81.7%) [64]. In contrast, this finding is higher than a study reported from Egypt (46.12%) [65] and Palestine (45.8%) [66]. The discrepancy might be due to differences in the socioeconomic status of participants. Moreover, there is a methodological variation between this study and the above-mentioned studies. Therefore, improving adherence to antihypertensive medications is crucial to decrease complications of uncontrolled hypertension.

In this systematic review and meta-analysis, knowledge on hypertension and the educational status of hypertensive patients were significant predictors of hypertensive self-care practice in Ethiopia. Hypertensive patients who attended formal education were approximately 3 times more likely to adhere to hypertensive self-care practice than those who did not attend formal education. This finding is supported by studies done in the Ashanti region of Ghana [50] and West Bengal, India [67]. A study conducted in Nepal showed that less-educated hypertensive patients were not adhering to hypertensive self-care practices like educated hypertensive patients [51]. This may be due to the fact that illiterate and less educated hypertensive individuals may not understand the benefits of self-care practices and the complications of poorly controlled and uncontrolled hypertension. Moreover, educated people can read and easily understand the recommended hypertensive treatment guidelines, which in turn improve self-care practice of hypertensive patients [68, 69]. Therefore, enhancing adult education has a significant impact to increase hypertensive self-care practices.

Finally, the results of this meta-analysis showed that hypertensive patients who had good knowledge about hypertension were 4 times more likely to practice self-care than those patients who had poor knowledge about hypertension. Similar findings were reported to form studies done in Israel [60]. Good knowledge about hypertension promotes patients to give more emphasis on hypertensive self-care practice. A randomized controlled trial conducted among hypertensive women showed that improving the knowledge of hypertensive patients through a participatory method of education could help hypertensive patients to practice better self-care and reduce disease complications [70].

## 5. Limitations of the study

The limitation of this meta-analysis in all regions of the country was not represented due to a lack of studies. Besides, the included studies reported the government health facility population and so this study does not consider hypertension patients who have follow-up in private clinics and home-dwelling people. Finally, all included studies have a cross-sectional study design, which shares the limitations of the cross-sectional study design.

## 6. Conclusion

In this study, more than half of the hypertensive patients had poor hypertensive self-care practice in Ethiopia. Participant's educational status and knowledge on hypertension were significantly associated with self-care practice among people living with hypertension in Ethiopia. Therefore, based on the evidence of this study, we recommend that programmers and policymakers should enhance the awareness of hypertensive patients on self-care practice domains and strengthen local programs working on noncommunicable diseases. Moreover, national population-based studies must be conducted to assess the risk factors that contribute to poor self-care practice among hypertensive patients in Ethiopia.

## Figures and Tables

**Table 1:** Characteristics of included studies to assess the level of self-care practice among hypertensive patients in Ethiopia.

No.	Author name	Publication year	Region	Sample size	Response rate (%)	Mean age	Overall self-care practice (%)	Salt reduction (%)	Physical activity (%)	Smoking cessation (%)	Moderate alcohol intake (%)	Weight management (%)	Drug adherence (%)	Quality
1	Kassahun et al. [40]	2020	Amhara	384	100	56	59.4	69.3	21.1	70.8	72.4	61.2	68	7
2	Hussen et al. [41]	2020	Harar	398	98.2	52	29.9	54.2	31	81.8	20.2	46.8	57.5	9
3	Ahmed and Teferi [44]	2020	Addis Ababa	384	—	57	51.5	47.2	55.7	82.1	71.5		59.3	8
4	Gebremichael et al. [27]	2019	Tigray	320	99.9	53.83	20.3	63.1	49.4	99.1	67.2	40.6	74.1	8
5	Nadewu and Geda [35]	2018	Harar	422	95	58.6	62.1	—	—	—	—	—	—	7
6	Abdulwahed and Seid [37]	2020	Oromia	322	98	—	44.7	70.5	76.7	97.2	95.3	81.7	40.4	7
7	Fetensa et al. [28]	2019	Oromia	222	99.9	44	68.92	45.9	52.5	98.2	80.5	50.9	—	7
8	Bacha and Abera [43]	2017	Addis Ababa	385	95.5	57.6	39.5	—	—	—	—	—	—	8
9	Niriayo et al. [46]	2019	Tigray	276	—	52.54	—	29	44.9	89.9	68.8	21.45	48.2	9
10	Tesema and Disasa [34]	2016	Oromia	130	—	—	—	80	—	98.5	85	—	—	7
11	Tibebu et al. [45]	2017	Addis Ababa	404	97	54	23	69.1	31.4	85.9	74.8	—	—	8
12	Andualem et al. [39]	2020	Amhara	301	99.9	51	23.6	76.4	39.9	89.7	94	—	—	8
13	Labata et al. [36]	2019	Oromia	341	96.8	54.35	—	30.5	44.9	93.5	88.3	56.9	61.9	8
14	Buda et al. [47]	2017	SNNPR	205	97.5	53.9	27.3	57.2	16.1	89.2	12.1	41.9	—	9
15	Angelo et al. [48]	2020	SNNPR	171	98.8	—	33.3	26.3	62.6	62.6	43.9	29.2	—	7
16	Bogale et al. [42]	2020	Harar	274	94	56.36	49.6	—	83.2	—	—	—	—	8
17	Ademe et al. [38]	2019	Amhara	309	99.9	58.8	49	—	—	70.8	72.4	61.2	68	9

SNNPR = South Nations Nationalities and Peoples Region.

**Table 2:** Subgroup analysis of the estimated pooled level of self-care practice among hypertensive patients in Ethiopia.

Variable	Subgroup	Included studies	Sample size	Estimate of overall hypertensive self-care	*I* ^2^, *p* value
Region	Amhara	3	994	43.99 (22.22, 65.75)	98.2%, ≤0.001
	Harar	3	1094	47.19 (27.20, 67.17)	97.9%, ≤0.001
	Addis Ababa	3	1173	37.95 (21.25, 54.65)	97.4%, ≤0.001
	SNNPR	2	376	30.02 (24.17, 35.88)	37%, 0.208
	Oromia	2	544	56.77 (33.03, 80.58)	97%, ≤0.001
	Tigray	1	320	20.3 (15.89, 24.71)	—
Total		14	4,501	41.55 (33.06, 50.05)	97.4, ≤0.001

SNNPR: South Nations Nationalities and Peoples Region.

**Table 3:** Pooled levels of adherence to the recommended self-care practice among hypertensive patients in Ethiopia.

Recommended lifestyle	Included studies	Sample size	Pooled level of good practice at 95% CI	*I* ^2^, *p*-value
Physical activity	13	4002	46.68 (35.03, 58.68)	98.6%, ≤0.001
Salt reduction	13	3852	55.03 (45.58, 65.03)	97.7%, ≤0.001
Cessation of smoking	13	3852	88.09 (83.99, 92.19)	97.1%, ≤0.001
Moderation of alcohol intake	13	3852	67.26 (52.26, 82.26)	99.4%, ≤0.001
Adherence to medications	7	2232	58.55 (50.26, 66.84)	94.7%, ≤0.001
Weight reduction	9	2446	47.90 (34.83, 60.97)	98.1%, ≤0.001

## Data Availability

All related data have been presented within the manuscript. The datasets supporting the conclusions of this article are available from the authors on request.

## Abbreviations

AOR: Adjusted odds ratio
BMI: Body mass index
CI: Confidence interval
PRISMA: Preferred reporting item for systematic review and meta-analysis
SNNPR: South Nation Nationalities and Peoples Region.
